# Oxidative Stress, Phytochemical Screening, and Antioxidant Activity on Microalgae (*Arthrospira platensis*) After Exposure to Glyphosate and Microplastics

**DOI:** 10.3390/jox15040106

**Published:** 2025-07-03

**Authors:** Dércia Santos, Edna Cabecinha, Jesús Gago, Sandra Mariza Monteiro, Ana Luzio

**Affiliations:** 1 Centre for the Research and Technology of Agro-Environmental and Biological Sciences, CITAB and Inov4Agro, Institute for Innovation, Capacity Building and Sustainability of Agri-Food Production, University of Trás-os-Montes and Alto Douro, Quinta de Prados, 5000-801 Vila Real, Portugal; edna@utad.pt (E.C.); smonteir@utad.pt (S.M.M.); aluzio@utad.pt (A.L.); 2 Centro Oceanográfico de Vigo, Instituto Español de Oceanografía (IEO-CSIC), Subida a Radio Faro 50, 36390 Vigo, Spain; jesus.gago@ieo.csic.es

**Keywords:** polyethylene terephthalate, polyamide, herbicides, blue-green microalgae, toxicity

## Abstract

The knowledge about the potential toxic effects of microplastics (MPs) combined with herbicides at lower trophic levels is still largely unknown. The present study aimed to evaluate the potential toxic effects of polyethylene terephthalate (PET) and polyamide (PA), isolated or combined with the pesticide glyphosate (GLY), on the microalgae *Arthrospira platensis*. For this, microalgae were exposed to control, GLY (3 μg/L), PET (0.5 and 1 mg/L), PA (0.5 and 1 mg/L), and the respective mixtures of each MP with GLY, for 12 days. The photosynthetic pigment content, phytochemicals, antioxidants, and enzymatic activity were determined. Cell growth was significantly enhanced on day 4 in the GLY+PA1 group (~80%), compared to the control. At day 12, biomass was significantly higher in the GLY (~25%) and GLY+PET0.5 (~26%) groups relative to the control. Significant effects on the enzymatic and detoxification mechanisms were observed, including increased SOD (PET0.5, *p* = 0.011) and CarE (GLY, PA and GLY+PA, *p* < 0.01), and decreased GST in combined exposures, which support stress-induced enzymatic activation and adaptive biochemical responses. Significant effects on phytochemicals and antioxidant activity were also observed, with PET0.5 significantly reducing total carotenoids (~65%), and flavonoids (*p* < 0.001) and ortho-diphenols (*p* < 0.05) being decreased in all exposure groups, in comparison to the control group. The decrease in flavonoids and ortho-diphenols, important antioxidant molecules, suggests the depletion of these key compounds under stress. DPPH scavenging activity, a measure of antioxidant potential, was inhibited in the GLY+PA groups, indicating compromised antioxidant defense. Results confirmed that combined stressors elicit distinct and sometimes deleterious responses not predicted by single exposures. Our findings highlight that the combined exposure to glyphosate and MPs significantly disrupts antioxidant defenses and enzymatic activity in *A. platensis*, indicating potential risks to primary producers in aquatic ecosystems and underscoring the ecological implications of co-contaminant stressors. In fact, the results indicate that MPs can modify herbicide toxicity, posing enhanced risks to microalgal physiology and potentially affecting primary productivity and nutrient cycling in aquatic ecosystems. In turn, negative effects of MPs on microalgae can have serious consequences for food webs, food security, and ecological health.

## 1. Introduction

The growing pollution of aquatic ecosystems caused by human-made contaminants has become a significant environmental issue, particularly with the increasing presence of microplastics in freshwater and marine habitats. Microplastics (MPs), defined as polymer particles smaller than 5 mm in diameter, are either formed from the breakdown of larger plastic materials (secondary MPs) or intentionally produced at a microscopic scale for specific applications, such as personal care products, synthetic textiles, and medicine (primary MPs) [1]. In freshwater ecosystems, MPs’ concentrations have been reported to range from 44.8 to 11,532 particles/m^3^, while in marine environments, levels vary between 0.23 and 267.2 particles/m^3^ [2]. MPs of different types, such as polyethylene terephthalate (PET) and polyamide (PA, also known as nylon), are widely found in aquatic ecosystems due to their extensive use in packaging materials, clothing, and the automotive industry [3,4]. Renzi et al. [5] reported that PET-based MPs constituted approximately 6.2–12.5% of total MPs in marine sediments and 25.0–29.5% in holothurians. Studies of coastal and freshwater sediments from South Korea, Japan, and the United States have found PET concentrations (on a dry weight basis) reaching up to 13,000 µg/g, 5.4 µg/g, and 10 µg/g, respectively [6]. In contrast, on Malaysian beaches, alarmingly high total MP concentrations (up to 57.72 mg/g) were reported in sediments, with PET comprising approximately 11–13% of this load [6]. Additionally, PA accounts for about 44.7% of the synthetic polymers discharged into the marine environment, further underscoring the prevalence of these materials in aquatic systems [7]. Research on MPs is expanding rapidly, with growing evidence that these particles induce toxic effects in fish, mussels, zooplankton, and phytoplankton at physiological, cellular, and molecular levels [4,8,9,10,11,12,13]. Furthermore, it has been suggested that MPs can act as vectors for other pollutants, such as heavy metals and organic pollutants, potentially influencing their bioavailability, persistence, and toxicity [14,15].

Concurrently, herbicide runoff from agricultural activities represents another diffuse, non-point source of pollution to aquatic ecosystems. Different studies have reported the detection of residues from approximately 31 different herbicides in over 768 water samples collected across 18 countries worldwide, with concentrations in surface waters ranging from 0.0001 to 79.02 μg/L [16]. When MPs co-occur with herbicides in these ecosystems, they may exert additive or synergistic toxic effects, leading to complex and potentially amplified impacts on aquatic organisms [17,18]. Among the most extensively used herbicides is glyphosate (N-(phosphonomethyl)glycine), the active ingredient in many broad-spectrum systemic herbicide formulations [19]. Glyphosate (GLY) exerts its effect by inhibiting the 5-enolpyruvylshikimate-3-phosphate synthase (EPSPS), a key enzyme in the shikimate pathway [20]. This enzyme catalyzes the conversion of shikimate-3-phosphate (S3P) and phosphoenolpyruvate (PEP) into 5-enolpyruvylshikimate-3-phosphate (EPSP), a critical step in the biosynthesis of aromatic amino acids, such as phenylalanine and tryptophan. Thus, inhibiting EPSPS disrupts the production of essential aromatic compounds, ultimately leading to plant death [20,21]. As the shikimate pathway is present in all plants, as well as in microalgae/cyanobacteria, bacteria, and fungi, GLY functions as a non-selective, broad-spectrum herbicide [20]. Due to surface runoff and mismanagement, GLY has been frequently detected in surface waters at trace levels, raising concerns about unintended effects on non-target aquatic organisms, including primary producers such as microalgae [20].

Microalgae, as key contributors to ecosystem balance, play a vital role in nutrient cycling and serve as the basis of aquatic food webs [22]. Previous research has reported that microalgae are sensitive to environmental pollutants, including MPs and herbicides, and even minor disturbances can lead to ecosystem-wide effects [22]. For instance, Yang et al. [23] found that exposure to polyethylene, polyamide, or polystyrene (13 μm and 150 μm) inhibited growth and caused oxidative stress in the microalgae *Chlorella pyrenoidosa*. Inhibition of photosynthetic efficiency, oxidative stress, and changes in the expression levels of photosynthesis-related genes (*psaB*, *psbC*, and *rbcL*) were also reported in microalgae exposed to single MPs [24,25,26]. Similarly, previous studies have shown that exposure to GLY decreases microalgae growth [19,27] and induces oxidative stress and metabolic alterations [27,28,29]. Current research indicates that the toxic effects of MPs and GLY on microalgae remain poorly understood. Furthermore, limited information on their combined impacts hinders a comprehensive understanding of the underlying mechanisms of MPs’ toxicity. Gaining insight into these interactions is essential, as disruptions at the base of the aquatic food web, where microalgae play a foundational role, can cascade through the ecosystem, threatening the higher trophic levels, the biodiversity, water quality, and ultimately impacting food security.

In this context, the present study aimed to evaluate the individual and combined effects of polyethylene terephthalate (PET) and polyamide (PA), with the herbicide GLY, on the microalga *Arthrospira platensis*. *A. platensis*, also known as Spirulina, is a filamentous, blue-green cyanobacterium commonly found in diverse environments, including alkaline lakes, ponds, freshwater, seawater, and thermal springs, and with a high adaptability to extreme environments [30,31]. It is well-suited for laboratory ecotoxicity studies due to its ease of cultivation, rapid growth rate, and high sensitivity to changes in physical and chemical environmental conditions [32]. More recently, it was reported that *A. platensis* is remarkably sensitive to aquatic pollutants, including pesticides and antibiotics [33]. This species is valued for its high nutrient content. As a result, it is widely used in the human health food industry and as a feed additive in aquaculture [30]. While its nutritional value and commercial use are well established, there is limited knowledge about how *A. platensis* responds to emerging contaminants such as MPs and GLY, particularly under co-exposure scenarios that reflect real-world conditions. To date, most ecotoxicological studies have focused on model green microalgae, leaving cyanobacteria, such as *A. platensis*, underrepresented in pollution research [34]. Furthermore, the mechanisms by which these contaminants affect microalgal physiology, including growth, pigment biosynthesis, and oxidative stress response, remain insufficiently explored. Addressing these gaps is crucial for understanding how contaminant mixtures affect the structure and function of primary producers in aquatic ecosystems. To this end, the present study evaluated multiple physiological and biochemical parameters, including growth, photosynthetic pigment content, phytochemicals, and enzymatic and antioxidant activities, to elucidate how combined stressors may compromise the health of microalgae, in particular, of this economically and ecologically important species.

## 2. Materials and Methods

### 2.1. Chemicals

The glyphosate-based formulation used in this study was Montana^®^ ASCENZA (ASCENZA Agro S.A., Setúbal, Portugal), containing 360 g/L of GLY (31% active ingredient). A stock solution of 36 mg GLY/L was prepared in Milli-Q water and used for the exposure treatments at a concentration of 3 µg/L. The experimental concentration of GLY was established based on the environmentally relevant concentrations reported in aquatic ecosystems (variation between 0.035 ng/mL and 96 μg/L) [35,36,37]. GLY concentrations vary depending on proximity to agricultural areas, seasonal application, and runoff patterns. While median values in Europe typically fall below 1 µg/L, peak concentrations of 2–5 µg/L have been observed in small water bodies near treated fields [38,39]. The selected concentration of 3 µg/L represents the upper range and was chosen to simulate a realistic high-exposure scenario within documented environmental levels, thereby enhancing the ecological relevance of the study. This concentration also exceeds the Portuguese drinking water limit by a factor of 30, as defined by the EU Directive 98/83/EC, which sets the threshold at 0.1 μg/L.

Virgin and irregularly-shaped polyethylene terephthalate (PET) and polyamide (PA) were purchased from local polymer materials suppliers and sieved to a size < 100 μm. Subsamples were analyzed for polymer confirmation using Fourier-transform infrared spectroscopy (FTIR)–attenuated total reflectance (FTIR–ATR) spectroscopy (Spectrum Two with Diamond ATR accessory, PerkinElmer, Waltham, MA, USA). The scan range used was 550 to 4000 cm^−1^, with a resolution of 8 cm^−1^, eight co-scans, and strong apodization. Results were compared with the microplastics/polymer library (FLOPP and FLOPP-e [40]) and in-house library and accepted for an HQI > 0.9 (hit quality index, Table S1). The spectral images obtained from FTIR–ATR of MPs are shown in Figure S1 (see Supplementary Materials). The concentrations used in this experiment, for both MPs, were 0.5 and 1 mg/L, and are considered environmentally relevant. According to previous studies, the mean abundances of MPs in aquatic ecosystems are ≤1 mg/L [41,42].

All the other analytical-grade reagents used were acquired from scientific retailers.

### 2.2. Microalgae Cultivation

Stock *Arthrospira platensis* (Spirulina) cultures were maintained in the aquatic facility of the University of Trás-os-Montes and Alto Douro (Vila Real, Portugal), as described by Zarrouk [43]. Microalgae were maintained at 30 ± 1 °C, on a 14:10 h light/dark cycle under fluorescent lighting (3200–3500 lux), in 250 mL Erlenmeyer flasks with Zarrouk’s medium. The nutritional composition of Zarrouk’s medium is presented in Table S2 (see Supplementary Materials). Cultures of *A. platensis*, which were kept in the exponential growth phase, were selected as the inoculum for all subsequent experiments. All the glassware and media used to culture algae were sterilized by autoclaving at 120 °C for 30 min before use.

### 2.3. Experimental Tests

All procedures were performed under aseptic conditions in a laminar flow chamber. The bioassays were performed in 250 mL Erlenmeyer flasks containing 150 mL of Zarrouk’s medium with different treatments: Control (medium without contaminants), GLY (3 µg/L), PET (0.5 and 1 mg/L), PA (0.5 and 1 mg/L), and respective mixtures of GLY with PET or PA (GLY+PET0.5, GLY+PET1, GLY+PA0.5, GLY+PA1). The experimental cultures were exposed for 12 days and maintained under the same conditions as stock *A. platensis*. All treatments were performed in triplicate using three independent culture flasks (biological replicates) for each condition. All the flasks were shaken three times per day and arranged randomly to prevent effects caused by variations in light irradiance.

Optical density (OD) was measured every 2 days, at a 565 nm wavelength in a microplate scanning spectrophotometer (PowerWave XS2, BioTek Instruments, Winooski, VT, USA) to determine the growth rate of microalgae. At the end of the bioassays, the dry biomass of the algae was determined by vacuum-filtering 4 mL from each group through a pre-weighed Whatman GF/C glass fiber filter and then drying it at 60 °C for 24 h. Samples for photosynthetic pigment content, phytochemicals, antioxidants, and enzymatic activities were also collected after 12 days of exposure.

### 2.4. Photosynthetic Pigment Content

The measurement of chlorophyll *a* (Chl*a*), chlorophyll *b* (Chl*b*), and total carotenoid content was carried out following the procedure outlined by Abbasi et al. [30], with slight modifications. For each treatment group, samples of 10 mL were centrifuged at 3000× *g* for 10 min (Labnet Prism R, Edison, NJ, USA), and the algal pellets were re-suspended in 80% acetone. The samples were incubated overnight at 4 °C and centrifuged again for 10 min at 3000× *g*. The absorbance of the supernatant was measured at 663 nm, 646 nm, and 470 nm using a microplate scanning spectrophotometer (PowerWave XS2, Bio-Tek Instruments, Winooski, VT, USA). Chlorophyll *a*, chlorophyll *b*, and total carotenoids concentrations were determined according to the following formulas:

Chl*a* = 12.7(A663) − 2.69(A646)

Chl*b* = 22.9(A646) − 4.68(A663)

Total carotenoids = [(1000(A470) − 1.82 Chl*a* − 85.02 Chl*b*]/198

where A663, A646, and A470 represent the absorbance values at 663, 646, and 440 nm, respectively. The photosynthetic pigment content was expressed in mg/L.

### 2.5. Phytochemicals, Antioxidant, and Enzymatic Activities Analysis

After 12 days of exposure, 50 mL of culture from each group was sampled and lyophilized for 96 h before analysis.

For the phytochemical and antioxidant analysis, 40 mg of *A. platensis* lyophilized extract was weighed and agitated at room temperature for 1 h in a MeOH/HCl extraction buffer. Samples were then centrifuged at 5000× *g* for 15 min at 4 °C (Labnet Prism R, Edison, NJ, USA), and the supernatant was stored at −20 °C until further analysis.

For the enzymatic activities, 10 mg of lyophilized extract was homogenized in a precooled 0.1 M potassium phosphate buffer (pH 7.5) using an automatic homogenizer (TissueLyser II, QIAGEN, Hilden, Germany) at 30 Hz for 1 min and 30 s. Following homogenization, the samples were centrifuged at 12,000× *g* for 15 min at 4 °C. The supernatant was collected and stored at −20 °C for subsequent analysis.

The protein concentration in all samples was estimated using Bradford’s method at 595 nm [44]. The following biochemical measurements were carried out using a microplate scanning spectrophotometer (PowerWave XS2, Bio-Tek Instruments, Winooski, VT, USA) set to 30 °C or a Varian Cary Eclipse (Agilent, Santa Clara, CA, USA) spectrofluorometer.

#### 2.5.1. Phytochemicals Analysis

The phytochemical analysis was performed using a previously established methodology described in Luzio et al. [45]. The total phenolic content (TPC) was determined using the Folin–Ciocalteu method at 725 nm, as outlined in Rodrigues et al. [46]. TPC results were expressed as milligrams of gallic acid equivalents per gram of extract (mg GAE/g dry weight, DW) with a calibration curve (y = 1.076x + 0.0598, R^2^ = 0.9999; blank). Total flavonoid content (TFC) was quantified using the aluminum chloride (AlCl_3_) complex method at 510 nm, also following Rodrigues et al. [46], and expressed as milligrams of catechin equivalents per gram of extract (mg CAE/g DW) with a calibration curve (y = 4.4144x + 0.1048, R^2^ = 0.9818; blank). Ortho-diphenols content (ODC) was assessed using a colorimetric method involving a complex reaction with sodium molybdate dihydrate at 370 nm, as described by Granato et al. [47]. ODC results were expressed as milligrams of gallic acid equivalents per gram of extract (mg GAE/g DW) with a calibration curve (y = 7.5196x + 0.0749, R^2^ = 0.9965; blank). All measurements were conducted in triplicate.

#### 2.5.2. Determination of Antioxidant Activity by Radical-Based Assays

The antioxidant activity was assessed according to a previously established methodology [45]. Radical scavenging activity (RSA) on the ABTS (2,2′-azino-bis (3-éthylbenzothiazoline-6-sulphonique)) radical was evaluated using the Trolox equivalent antioxidant capacity (TEAC) assay at 734 nm, as described by Rodrigues et al. [46]. Results for ABTS RSA were expressed as milligrams of Trolox equivalents per gram of extract (mg TE/g dry weight, DW), with a calibration curve (y = −0.7885x + 0.8239, R^2^ = 0.9918; blank). RSA on the DPPH (2,2-diphenyl-1-picrylhydrazyl) radical was determined according to Rodrigues et al. [46] and expressed as milligrams of Trolox equivalents per gram of extract (mg TE/g DW), with a calibration curve (y = −0.8435x + 0.8809, R^2^ = 0.9862; blank).

#### 2.5.3. Enzymatic Activities Determination

Standardized protocols, with slight modifications, were used to determine oxidative stress-related biomarkers [9]. Samples were analyzed in duplicate. SOD activity was determined by measuring the inhibition of the reduction of nitroblue tetrazolium, at 560 nm, following the method of Durak et al. [48]. Superoxide dismutase (SOD) activity was quantified using a standard curve of SOD (0–60 U/mL), which was used to express its activity as U/mg protein. Glutathione reductase (GR) activity was measured using the method described by Massarsky et al. [49] at 340 nm. The NADPH extinction coefficient (6.22 mM^−1^ cm^−1^) was used to estimate the activity of GR. Glutathione S-transferase (GST) activity was determined following the method of Habig and Jakoby [50], at 340 nm, using 1-chloro-2,4-dinitrobenzene (CDNB) as substrate, and expressed as µmol/min.mg protein. The carboxylesterase (CarE) activity, expressed as nmol 4-nitrophenol/min.mg, was assessed by measuring the reaction product of *p*-nitrophenol at 405 nm [51]. The reduced (GSH) and oxidized (GSSG) glutathiones were evaluated following the methodology of Gartaganis et al. [52], at 320/420 nm (excitation/emission wavelengths), and expressed as µmol/mg protein. Reactive oxygen species (ROS) content was measured following the method described by Deng et al. [53] at 485/530 nm (excitation/emission wavelength). A 2′,7′-dichlorofluorescein (DCF) standard curve (0–6.25 nM) was used to estimate ROS levels, which were expressed as µmol DCF/mg protein. The biochemical levels and activities were normalized to total protein.

### 2.6. Statistical Analysis

The analysis of variance (ANOVA) statistical tests was performed using Prism 10.3.1 software (GraphPad Software, Inc., Boston, MA, USA). Homoscedasticity and normality were assessed using the Brown–Forsythe and Kolmogorov–Smirnov tests, respectively. The results from the different treatments were then compared using one-way ANOVA followed by Tukey’s post hoc test. Statistical differences were considered significant when *p* < 0.05.

To detect correlations between the data, a multivariate analysis was employed. Response data have a gradient of 0.1 SD units long, so the linear and unimodal methods were applied. A constrained ordination, Canonical Correspondence Analysis (CCA), was used to extract and summarize the variation in the response variables that the explanatory variables can explain. The CCA was carried out using CANOCO 5 (version 5.15) [54].

## 3. Results

### 3.1. Cell Growth and Photosynthetic Pigment Contents

**Figure 1:**
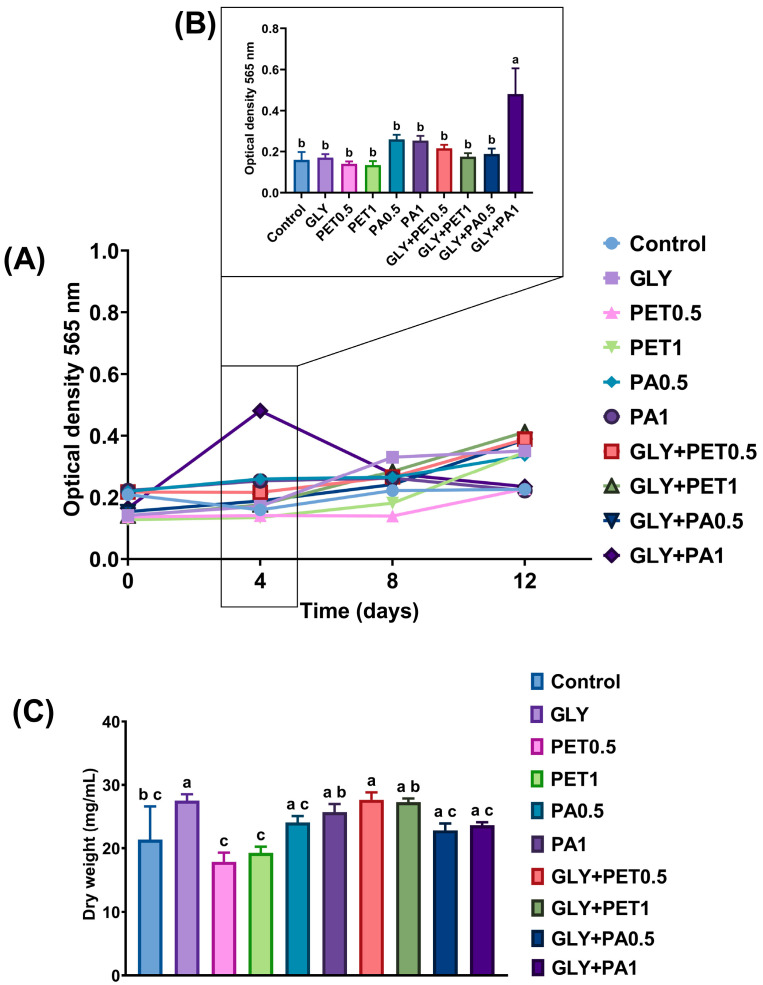
Cell growth as optical density as a function of exposure time (**A**,**B**) and dry weight (**C**) of *Arthrospira platensis* exposed for 12 days to polyethylene terephthalate (PET) and polyamide (PA) microplastics alone or combined with glyphosate (GLY). Data are expressed as mean ± S.D. of three replicates. Different lowercase letters indicate significant differences (*p* < 0.05).

*A. platensis* cell growth in response to GLY and MPs exposure is presented in Figure 1. Overall, no significant differences were observed in growth between the GLY and MPs on most sampling days (Figure 1A). The exception occurred on day 4, where microalgae exposed to the GLY+PA1 mixture exhibited a significant increase in growth compared to both the control and all other exposure groups (*p* < 0.0001, Figure 1B).

Mean dry biomass after 12 days of exposure (Figure 1C) was significantly higher in microalgae exposed to GLY alone and GLY+PET0.5, compared to the control group (*p* < 0.05). Additionally, both GLY+PET groups showed significantly greater biomass than their respective single PET groups (*p* < 0.01), suggesting a synergistic interaction between these pollutants.

**Figure 2:**
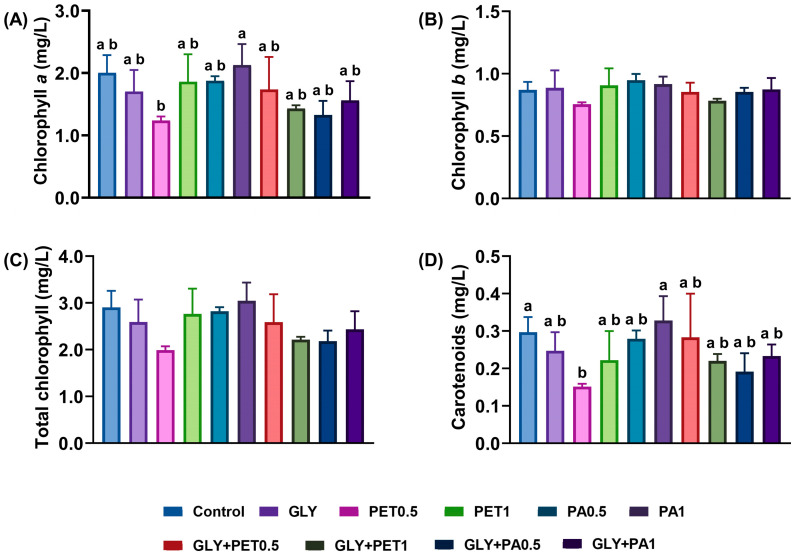
Photosynthetic pigments—chlorophyll a (**A**), chlorophyll b (**B**), total chlorophyll (**C**), and carotenoids (**D**)—of *Arthrospira platensis* exposed for 12 days to polyethylene terephthalate (PET) and polyamide (PA) microplastics alone or combined with glyphosate (GLY). Data are expressed as mean ± S.D. of three replicates. Different lowercase letters indicate significant differences (*p* < 0.05).

The mean concentrations of photosynthetic pigments—chlorophyll *a*, chlorophyll *b*, and total carotenoids—are shown in Figure 2. No significant differences were observed in Chl*a*, Chl*b*, or total chlorophyll between exposure groups and the control (Figure 2A–C, *p* > 0.05). However, a significant reduction in total carotenoids was observed in the PET0.5 group compared to the control (*p* = 0.041, Figure 2D).

### 3.2. Effects on Phytochemicals and Antioxidant Activity

**Figure 3:**
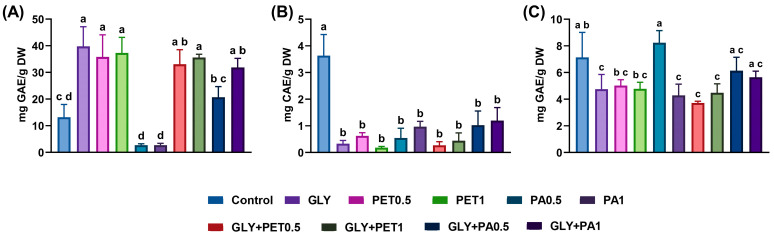
Phenolic compounds—total phenols (**A**), flavonoids (**B**), and ortho-diphenols (**C**)—of *Arthrospira platensis* exposed for 12 days to polyethylene terephthalate (PET) and polyamide (PA) microplastics alone or combined with glyphosate (GLY). Data are expressed as mean ± S.D. of three replicates. Different lowercase letters indicate significant differences (*p* < 0.05).

Figure 3 presents the concentrations of phenolic compounds—total phenols, flavonoids, and ortho-diphenols—in *A. platensis* following exposure to GLY and MPs.

Total phenols (Figure 3A) were significantly increased in groups exposed to GLY and PET alone, GLY+PET, and GLY+PA1 compared to the control (*p* < 0.01). Notably, the GLY+PA mixtures resulted in higher phenol levels than PA alone, but lower than GLY alone, indicating a potential antagonistic interaction between these stressors. Significant differences were observed between the individual GLY and PA treatments and their mixtures (*p* < 0.01). Flavonoid content (Figure 3B) was significantly decreased in all exposed groups compared to the control (*p* < 0.0001). For ortho-diphenols (Figure 3C), a significant decrease was observed in microalgae exposed to GLY, PA1, and both GLY+PET groups (*p* < 0.05).

**Figure 4:**
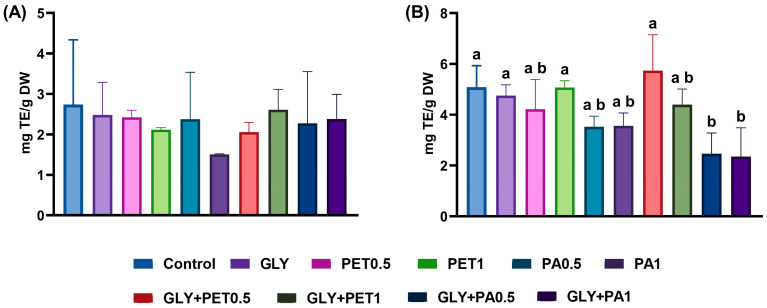
Antioxidant activity—(**A**) ABTS and (**B**) DPPH methods—of *Arthrospira platensis* exposed for 12 days to polyethylene terephthalate (PET) and polyamide (PA) microplastics alone or combined with glyphosate (GLY). Data are expressed as mean ± S.D. of three replicates. Different lowercase letters indicate significant differences (*p* < 0.05).

The antioxidant capacity of *A. platensis* was assessed using the ABTS and DPPH free radical-scavenging assays, as shown in Figure 4. For the ABTS assay (Figure 4A), no significant differences in antioxidant activity were observed across exposure groups (*p* > 0.05), although a slight decreasing trend was noted in both the PET and PA treatments. In contrast, the DPPH assay (Figure 4B) revealed a significant decrease in antioxidant activity in microalgae exposed to GLY+PA mixtures (*p* < 0.05), suggesting an antagonistic effect on the DPPH radical-scavenging capacity under combined GLY and PA stress.

### 3.3. Effects on Enzymatic Biomarkers

**Figure 5:**
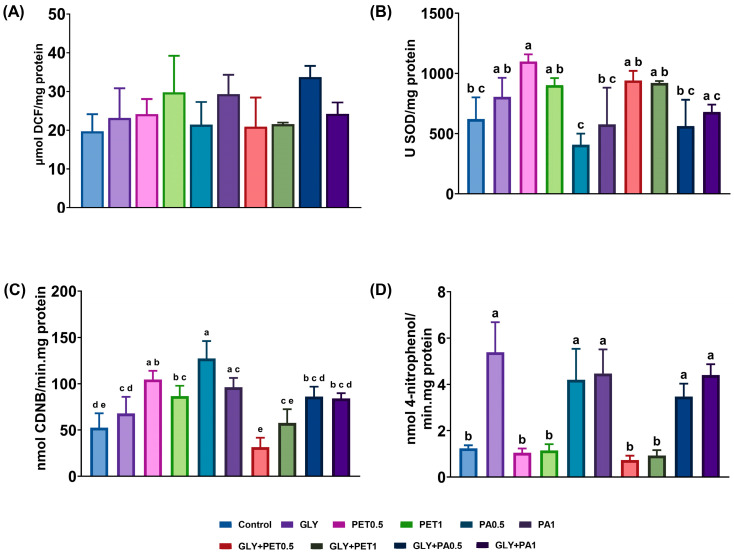
(**A**) Reactive oxygen species (ROS) levels, (**B**) superoxide dismutase (SOD), (**C**) glutathione S-transferase (GST), and (**D**) carboxylesterase (CarE) activities of *Arthrospira platensis* exposed for 12 days to polyethylene terephthalate (PET) and polyamide (PA) microplastics alone or combined with glyphosate (GLY). Data are expressed as mean ± S.D. of three replicates. Different lowercase letters indicate significant differences (*p* < 0.05).

Figure 5 illustrates the effects of GLY and MPs exposure on ROS levels, SOD, and detoxification enzyme responses in *A. platensis*. ROS levels (Figure 5A) remained unchanged in all exposed groups compared to the control (*p* > 0.05). In contrast, SOD activity (Figure 5B) significantly increased in the PET0.5 group relative to the control (*p* = 0.011). A response of the detoxification enzyme GST was also observed (Figure 5C), which showed higher activity in all groups exposed to MPs and GLY+PA0.5 (*p* < 0.01). Interestingly, GLY+PET0.5 and GLY+PA0.5 groups showed a significant decrease in GST activity compared to their respective individual exposures (GLY and MPs), suggesting a possible antagonistic interaction under combined stress conditions. For CarE activity (Figure 5D), a significant increase was observed in microalgae exposed to GLY, PA, and GLY+PA mixtures (*p* < 0.01).

**Figure 6:**
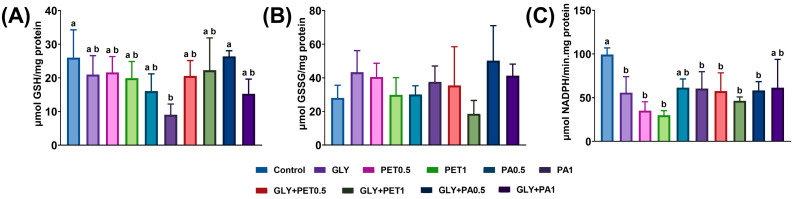
(**A**) Reduced glutathione (GSH), (**B**) oxidized glutathione (GSSG), and (**C**) glutathione reductase (GR) activity of *Arthrospira platensis* exposed for 12 days to polyethylene terephthalate (PET) and polyamide (PA) microplastics alone or combined with glyphosate (GLY). Data are expressed as mean ± S.D. of three replicates. Different lowercase letters indicate significant differences (*p* < 0.05).

Glutathione content and GR activity in *A. platensis* exposed to GLY and MPs are shown in Figure 6. GSH levels (Figure 6A) decreased in PA1-exposed microalgae, in comparison to the control group (*p* = 0.023), whereas GSSG levels (Figure 6B) remained stable across all treatment groups (*p* > 0.05). In contrast, GR activity (Figure 6C) was significantly inhibited in microalgae exposed to GLY, PET, PA1, GLY+PET, and GLY+PA0.5 (*p* < 0.05).

### 3.4. Canonical Correspondence Analysis of Photosynthetic Pigment Profiles, Phytochemicals, Antioxidants, and Enzymatic Activities

**Figure 7:**
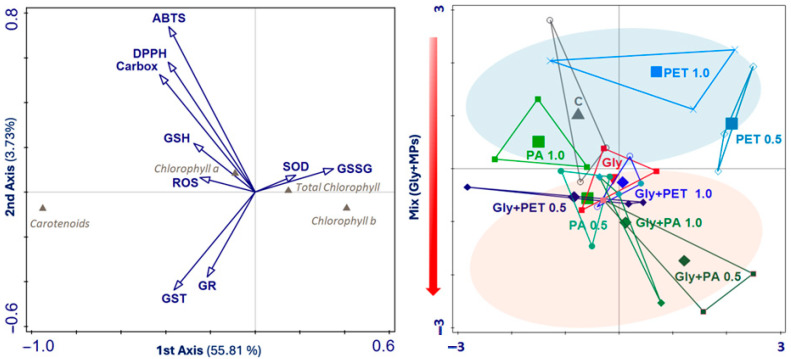
Canonical Correspondence Analysis (CCA) showing the total variance explained, for the first two axes, for the different treatments [Control (C), Glyphosate (GLY), and microplastics (MPs—PET and PA, at different concentrations 0.5 and 1 mg/L), and the mixture of both (GLY+PET, GLY+PA)] in *Arthrospira platensis* exposed for 12 days. The analysis included the photosynthetic pigments (chlorophyll *a*, *b*, total, and carotenoids), phytochemicals, antioxidants, and enzymatic activities. The arrows show the mixture gradient presented in the different plots and axes. Data from three independent replicates.

Canonical Correspondence Analysis (CCA) was used to identify which biochemical responses in *A. platensis* were most influenced by exposure to single GLY and MPs or their mixtures (GLY+PET, GLY+PA) (Figure 7). The analysis accounted for 59.7% of the total variance (180.000), with the first two axes explaining 59.5% of the variation (F = 27.8, *p* = 0.007; Table 1).

SOD activity and GSSG content were positively correlated with Axis 1 and primarily associated with the PET0.5 and PET1 treatments. In contrast, GST and GR enzymatic activities were negatively correlated with both Axis 1 and Axis 2, showing an inverse relationship with PET exposure. On Axis 2, antioxidant activities (ABTS, DPPH), ROS levels, GSH content, and CarE activity were positively correlated but negatively associated with the mixture groups (GLY+PET and GLY+PA), suggesting a distinct biochemical response under combined stress conditions.

## 4. Discussion

The present study investigated the effects of environmentally relevant concentrations of GLY and two types of microplastics, PET and PA, both individually and in mixtures, on the microalgae *A. platensis*. The findings offer valuable insights into how *A. platensis* responds to these two environmental stressors, both alone and in combination.

Growth analysis indicated that exposure to GLY and MPs did not significantly affect *A. platensis* cell proliferation over the 12-day period, apart from a pronounced increase on day 4 in the GLY+PA1 group. This unexpected growth stimulation could reflect an initial stress-induced response that has led to temporary overcompensation in growth. Furthermore, an accumulation of biomass was observed in the GLY and GLY+PET0.5 treatments after 12 days of exposure, suggesting stimulated nutrient assimilation. A lack of consensus has been observed among studies regarding the growth of microalgae. Some studies have reported a growth-stimulating effect on algae exposed to individual MPs [55,56,57,58] or to GLY [59,60], while others have shown inhibitory effects [10,27,28,30,61,62]. In turn, these differences in the individual effects of GLY and MPs appear to be dose-dependent and related, for example, to the type and size of polymer, specific GLY formulation used, and microalgae species [27,63].

Photosynthetic pigments, including chlorophylls (chlorophyll *a*, *b*, and total chlorophyll) and carotenoids, are vital indicators of algal photosynthetic capacity and their physiological condition. Therefore, changes in pigment content are acknowledged as integral to the organism’s defense and acclimation strategies when facing stressful conditions [1]. In the present study, photosynthetic pigment analysis revealed stable levels of chlorophyll *a*, chlorophyll *b*, and total chlorophyll in all exposed groups. However, the significant reduction in total carotenoids in the PET0.5 group is noteworthy. Carotenoids are found in thylakoid membranes and play a crucial role in light harvesting and energy transfer during photosynthesis [64]. Additionally, carotenoids can protect photosystems from oxidative damage by directly scavenging ROS and reducing cell damage, quenching chlorophyll triplet states, or indirectly, through thermal dissipation of excess light energy [64]. It has been reported that increasing carotenoid content is a common response of microalgae to pollutants to protect them from oxidative stress [65]. However, carotenoid depletion in *A. platensis* exposed to PET may also reflect an oxidative stress response. Zamani-Ahmadmahmoodi et al. [65] have shown that with increasing exposure time (>10 days) to pollutants, the level of total carotenoids of exposed marine microalgae (*Nannochloropsis oculata*) declined compared to the control, indicating that chronic stress may exceed the total antioxidant capacity. Nevertheless, the lack of changes in chlorophylls, despite the reduction in carotenoids, suggests a subtle yet non-functionally disruptive impact on the photosynthetic apparatus. Similarly, previous studies have found no significant effects of MPs or GLY on the microalgae chlorophyll content [28,66].

Most research on microalgal responses to GLY or MPs has primarily focused on changes in cell growth, photosynthetic pigments, and enzymatic biomarkers [23,25,27,28,30,55,57] while significantly neglecting the evaluation of phenolic composition and antioxidant activity. Phenolic compounds, including total phenols, flavonoids, and ortho-diphenols, are an abundant class of secondary metabolites and key indicators of oxidative stress and defense responses in microalgae [67,68,69]. *A. platensis* is known to produce a diverse range of secondary metabolites, including phenolics, carotenoids, phycobiliproteins, and polysaccharides, many of which are regulated in response to environmental stressors such as UV radiation, heavy metals, herbicides, and MPs [68,70,71]. In the present study, the increase in total phenolic content observed under exposure to GLY, PET, and their combination (GLY+PET) suggests the activation of general stress–response pathways in *A. platensis*. However, flavonoid levels were significantly reduced in all exposed groups, as well as a notable decline in ortho-diphenols in the GLY, PA1, and GLY+PET treatments. This discrepancy may reflect a metabolic shift in phenolic biosynthesis, where the production of certain phenolic subclasses is prioritized over others [72]. Alternatively, it may indicate a suppression of key biosynthetic pathways. For instance, it is well known that GLY blocks the shikimic acid pathway [73,74], resulting in a reduction in the synthesis of aromatic amino acids, such as phenylalanine [20,27]. In higher plants and microalgae, phenylalanine serves as a critical precursor for the phenylpropanoid pathway, the main route for the biosynthesis of flavonoids and other phenolic compounds such as ortho-diphenols [75,76,77]. Therefore, the disruption of this pathway may underlie the observed reduction in specific phenolic compounds in *A. platensis* [78]. Moreover, environmental stressors such as UV-B radiation and heavy metal exposure have also been reported to induce oxidative stress in *A. platensis*, triggering the modulation of secondary metabolite production as part of the cellular defense strategy [70,79]. For example, carotenoid biosynthesis can increase under high light or oxidative stress, whereas phycocyanin and flavonoid levels may decrease under sustained toxic pressure [71].

As stated above, carotenoids and phenolic compounds are among the most relevant non-enzymatic antioxidant molecules in microalgae [1,67]. The ABTS and DPPH assays provided a broad measure of antioxidant capacity. While ABTS activity remained unchanged, a significant reduction in DPPH scavenging was observed in the GLY+PA groups. Since phenolic compounds are the most reactive and important ones that react easily with DPPH [80], the observed depletion of both flavonoids and ortho-diphenols may explain the reduced DPPH activity. Moreover, the observed reduction in DPPH radical-scavenging activity indicates that the combined exposure to GLY and PA may impair the antioxidant defense capacity of *A. platensis*. In a previous study, *Spirulina* exposed to high concentrations of polystyrene MPs showed a decrease in phycocyanin, a pigment–protein complex with antioxidant activity [81]. Nevertheless, there is a clear need for comprehensive research on the non-enzymatic antioxidant responses (secondary metabolites) of microalgae to MPs, both individually and in combination with other environmental contaminants, as the current literature provides limited insight into these responses.

Enzymatic responses provided critical insights into the internal oxidative and detoxification mechanisms of *A. platensis* in response to exposure to GLY and MPs. While ROS levels remained stable, SOD activity, an enzyme of the first line of defense, was significantly elevated in PET0.5-exposed cells, indicating a compensatory upregulation of primary antioxidant defense. It has been stated that in response to diminished antioxidant defenses, as observed in this study, organisms often modulate SOD activity as a compensatory mechanism to counteract oxidative stress [82]. Similarly, previous studies have reported elevated levels of SOD following exposure to MPs [10,55,82]. GST and CarE are two enzymes crucial in detoxifying xenobiotics such as environmental contaminants. GST activity increased in MPs and GLY+PA0.5 exposures, as well as CarE activity in GLY, PA, and GLY+PA groups, pointing to the activation of the detoxification system. Microalgae exposed to different herbicides [83] and MPs [84] have also shown increases in detoxification enzyme activity, supporting our findings. The glutathione reduced (GSH) content, which is also involved in antioxidant defense, declined in the PA1-exposed microalgae, while the oxidized form (GSSG) remained stable, suggesting that GSH was consumed without effective recycling. The observed inhibition of glutathione reductase (GR), a key enzyme responsible for regenerating reduced glutathione (GSH) from its oxidized form (GSSG), across nearly all treatments may account for the depletion of GSH. A reduced capacity to regenerate GSH could impair the cell’s ability to maintain redox homeostasis, particularly under stress conditions. In fact, depletion of GSH may have compromised the microalgae’s ability to neutralize free radicals, as reflected by the observed diminished DPPH scavenging activity. Overall, our findings suggest a broader oxidative stress response, where both enzymatic and non-enzymatic defense components were disrupted in *A. platensis* upon exposure to MPs and GLY.

To the best of our knowledge, research on the effects of co-contamination by MPs and GLY in microalgae remains limited and underexplored [85,86]. The CCA analysis offered an integrated view of the biochemical responses across treatments, with our results suggesting that microalgae responded differently to the single pollutants or the combination of both, confirming that combined stressors elicit distinct and sometimes deleterious responses that are not predicted by single exposures. In the biochemical responses of *A. platensis*, it was observed that mostly antagonistic interactions occurred between microplastics, in particular PA, and GLY. These antagonistic effects are likely due to reduced bioavailability or toxicity of GLY when co-exposed with MPs, which could be the result of a strong adsorption capacity of MPs for GLY. The physicochemical characteristics of MPs, including the type of polymer and the chemistry of their functional groups, influence their sorption capacity, which ultimately affects the bioavailability and toxicity of pollutants [87]. While hydrophobicity is generally considered a key factor governing the sorption of pollutants to MPs, GLY’s hydrophilic and polar nature suggests that additional mechanisms, such as pore-filling, electrostatic interactions, hydrogen bonding, and π–π interactions, may also play significant roles [87]. For instance, it has been reported that PA has a high adsorption capacity for hydrophilic pollutants, due to its porous structure and the formation of hydrogen bonds [88,89].

Similarly, recent research has shown the multifaceted interactions between MPs and GLY in different species. Yu et al. [90] showed that MPs enhanced the toxicity and bioavailability of GLY in the floating plant *Salvinia cucullata*. On *Daphnia magna* exposed to GLY combined with polyethylene microbeads and polyethylene terephthalate/polyamide (PET/PA) fibers, additive and synergistic effects were observed, depending on the chemical formulation of GLY [11]. More recently, antagonistic effects of MPs toward the toxicity of GLY were observed in *Limnospira maxima* [86]. Nevertheless, knowledge regarding the adsorption of pesticides to MPs remains limited, highlighting the need for further studies focused on gaining mechanistic insights, including adsorption kinetics. Our findings, along with those of previous studies, underscore the importance of assessing how plastic particles interact with waterborne pollutants, particularly pesticides.

## 5. Conclusions

Overall, the exposure of microalgae *A. platensis* to environmentally relevant concentrations of GLY and both microplastics, PET and PA, triggered notable biological responses, affecting mostly phytochemicals and biochemical defenses. Our findings underscore the ecotoxicological relevance of combined GLY and MPs exposure, demonstrating that co-contamination can lead to synergistic or antagonistic interactions that affect physiological and detoxification pathways in *A. platensis*. This study emphasizes the importance of considering pollutant mixtures in environmental risk assessments, as their combined effects may diverge significantly from those of individual compounds, potentially compromising the stability of aquatic ecosystems and food webs.

While this study offers valuable insights into the individual and combined effects of MPs and GLY on *A. platensis*, some limitations should be noted. Only one environmentally relevant concentration of GLY was tested, limiting the assessment of potential dose-dependent effects. Additionally, the use of pristine MPs does not fully capture the complexity of environmental plastics, which are typically weathered and often more toxic due to the adsorption of pollutants. Therefore, future studies should address these factors to reflect real-world exposure scenarios better and strengthen ecological relevance.

## Figures and Tables

**Table 1:** Summary of canonical correspondence analysis (CCA) results for different treatments (GLY, PET, and PA), and their effect on the *Arthrospira platensis* responses.

	All Variables	
	Axis 1	Axis 2
**Total variation**	180.000	
**Explanatory variables account for**	59.7%	
**Explained fitted variation (cumulative)**	55.8%	13.71%
**Permutation Results**		
**On the first axis**	F = 27.8, *p* = 0.007	
**On all axes**	F = 3.6, *p* = 0.006	

## Data Availability

The original contributions presented in this study are included in the article/Supplementary Materials. Further inquiries can be directed to the corresponding author(s).

## Supplementary Materials

The following supporting information can be downloaded at https://www.mdpi.com/article/10.3390/jox15040106/s1, Table S1—Polymers used in the present study and the hit quality index (HQI) of FTIR-ATR spectroscopy identification, Figure S1—Images and FTIR-ATR spectra of (a) Polyamide and (b) Polyethylene terephthalate, Table S2—Zarroouk’s medium composition.
