# Supplementary material for: Oxidative Stress, Phytochemical Screening, and Antioxidant Activity on Microalgae (Arthrospira platensis) After Exposure to Glyphosate and Microplastics
Source: J Xenobiot. 2025 Jul 3;15(4):106. doi: 10.3390/jox15040106 (PMC12286146; doi:10.3390/jox15040106)
Supplement: Supplementary file 1 [file jox-15-00106-s001.zip › jox-3705420-supplementary.pdf]

Supplementary materials: Oxidative Stress, Phytochemical Screening, and Antioxidant Activity on Microalgae (*Arthrospira platensis*) After Exposure to Glyphosate and Microplastics

Dércia Santos, Edna Cabecinha, Jesús Gago, Sandra Mariza Monteiro and Ana Luzio

Table S1. Polymers used in the present study and the hit quality index (HQI) of FTIR-ATR spectroscopy identification.

**Polymer sample**	Density (g/cm³)	**Identified as**	**HQI**
PA	1.14	Polyamide	0.9434
PET	1.38	Polyethylene terephthalate	0.9896

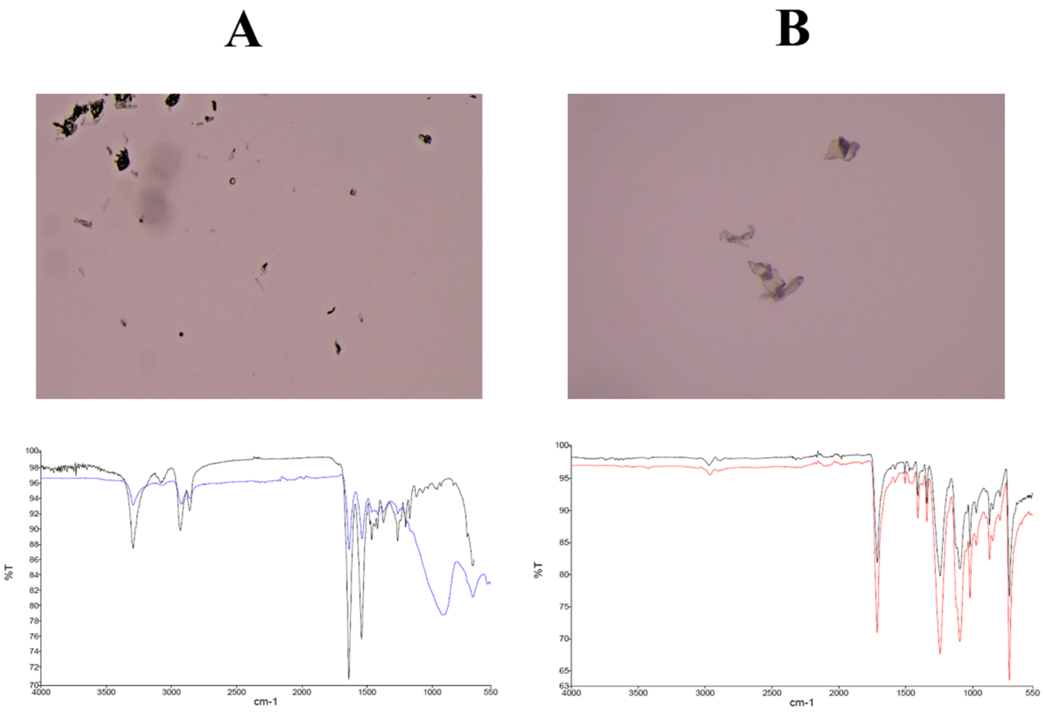

Figure S1. Images and FTIR-ATR spectra of a) Polyamide and b) Polyethylene terephthalate.

Table S2. Zarroouk's medium composition.

Components		g/L
Sodium nitrate	$NaNO_3$	2.5
Sodium bicarbonate	$NaHCO_3$	16.8
Sodium chloride	NaCl	1.0
Potassium sulfate	K_2SO_4	1.0
Dipotassium phosphate	K_2HPO_4	0.5
Ethylenediaminetetraacetic acid	EDTA	0.08
Calcium chloride dihydrate	$CaCl_2.2H_2O$	0.05
Iron(II) sulfate heptahydrate	$FeSO_4.7H_2O$	0.01
Magnesium sulfate heptahydrate	$MgSO_4.7H_2O$	0.2
**Trace elements mixture - 10 mL/L**		
Boric acid	H_3BO_3	0.003
Manganese(II) chloride tetrahydrate	$MnCl_2.4H_2O$	1.81
Zinc sulfate heptahydrate	$ZnSO_4.7H_2O$	0.222
Molybdenum trioxide	MoO_3	0.015
Copper sulfate pentahydrate	$CuSO_4.5H_2O$	0.074
